# Causes of Insanity*From report of Dr. C. L. Gregory, Superintendent of the North Texas Insane Hospital, Terrell, Texas.

**Published:** 1910-05

**Authors:** 


### TEXAS MEDICAL JOURNAL.

Established July, 1885

F. E. DANIEL, M. D., - - - - - - Editor, Publisher and Proprietor

Published Monthly.—Subscription $1.00 a Year.

VOL. XXV AUSTIN, MAY, 1910. No. 11

The publisher is not responsible for the views of the contributors.

### Original Articles.

For Texas Medical Journal.

### Causes of Insanity.*

Insanity is increasing out of proportion to the general population, not only in Texas and the United States, but in many countries of Europe. We take 1850 as the basis for our investigation. The statistical increase is startling. England's registered insane numbered at that time only 30,000. Thirty years later, with a population but 25 per cent greater, there were 70,000, an increase of over 100 per cent. Today the estimated increase is over 25 per cent above that of 1850, and England has an annual admission of over 21,000. The prevailing ratio of insanity over the continent of Europe is 1 to 300. A high insane ratio is self-serving and means a nervously bankrupt nation.

Take California, for instance, entering the Union in 1849. Its settlers were strong, hardy and healthy. In the year 1851, with a population of 130,000, there were only 6 registered insane, or a ratio of 1 to 21,000. In 1860 the ratio was 1 to 1000, and in 1870, California had one insane person tabulated for every 500 of the population. So rapidly has insanity increased in California that the registered insane number is 6555, or 1 to 240. In 1860 Texas had only 50 entries for her insane, or 1 to 12,800 of population. Today we have over 4000 insane, or about a ratio of 1 to 450.

New York's insane ratio to sane population is 1 to 330; Mass-

*From report of Dr. C. L. Gregory, Superintendent of the North Texas Insane Hospital, Terrell, Texas.

achusetts 1 to 370. These ratios are taken from the census of 1890, the 1900 census not being available. Suffice it to say, that the insane ratio is much greater now.

In discussing the problem and increase of insanity in California, the State Commission in Lunacy says: "In order to present this intelligently, we will divide the causes of insanity into two distinct classes—the predisposing and the exciting cause. To illustrate: Not many years ago consumption was considered to be hereditary, the disease being transmitted from the parent to the child. Now we know that the disease is not transmitted, but is produced by tuberculous bacilli; and that what is transmitted to the child is a weak physical organization endowed with a moderate amount of vital force and a lessened resistance to the encroachment of disease. Now the same principle appears in the predisposing causes of mental diseases. A very large percentage of mental diseases is the result of a weak, unstable nervous organization received from ancestors or acquired by dissipation or wasting diseases by the patient himself. It is a general law that everything that interferes with the normal action of the nervous system in either of the parents has a deleterious effect on the mentality of the child, and this condition is more marked and intensified in the child than in the parents. Alcohol, for instance, has a more deleterious effect upon the child than upon the person who drinks it. It poisons the germs in its development, and such a germ is not able to produce a healthy, normal individual, and the result is a weakened nervous organization, unstable in its action, a weak will, and a consequent loss of self-control. This condition of the nervous system has been termed neuropathic, and a person so afflicted is said to have a neuropathic constitution. Many persons have received from their ancestors or have acquired for themselves a weak, unstable nervous organization, unable to withstand the trials of life. From this class of weaklings come in a large measure the criminals, the paupers and the insane. The predisposing cause is generally the weak neuropathic organization and the exciting causes, the exigencies of human life, alcoholic indulgence being perhaps the most frequent, but by no means the only one. See table 'G' of Dr. King's report, Superintendent of the Mendocino Hospital, to the California State Commission in Lunacy, under the head of 'Causes.' Of the 253 commitments in 1908, 100 males and 14 females used alcohol to excess. From this table we learn that 45 per cent of those committed to this hospital used alcohol to excess. In a large percentage of cases one or both parents used alcohol to excess.

In most of these cases alcohol was probably an exciting cause, the predisposing cause being heredity. At this point it is pertinent to ask: What was the cause of this abnormal heredity? In order to get the real facts we must ascertain the history of the patient's ancestors in each case. This is a very difficult matter, and in many cases can not be done."

We can get much information from relatives and we find in the ancestor a history of wasting disease, alcoholism, epilepsy or insanity in 80 per cent of all cases. "All of these things reduce the tone of the system and the germ which is the father of the child, having been affected by the abnormal condition which produced it, develops into an imperfect child, with a result of imperfect manhood, with imperfect self-control, a weak, unstable nervous organism, a rich soil for the development of criminals and the insane. This condition is responsible for much of our crime and insanity. The causes are deeply rooted in the human family, in many cases reaching back to the second or third generation. Every healthy normal man who from any cause, either from alcoholic debauchery of other excesses, lowers the tone of his vital forces and afterwards propagates the species is preparing the soil which will bring forth a class of degenerates, many of whom will be insane or criminals, feeble-minded or idiotic."

Every unbiased man who investigates as to the cause of the widespread growth of insanity in this country must admit the foregoing causes are responsible for a large per cent of our insanity—the bane of civilization. The belief that insanity is not on the increase is not responsive to the immutable and unchallengeable figures tabulated in the Bureau of Statistics in the United States. I assert if insanity is to be diminished in Texas, it must be by prevention and not by cure. When the physicians of Texas put on the armor of bravery and courage and instruct the people as to the value and importance of good morals, hygiene, and at the same time calling attention to the ills following the marriage of people into families of lunatics, drunkards and criminals, the sooner they sound the alarm and teach the people the causes of insanity, the quicker we will begin to prevent its increase.

Preventive measures will prove more humane and less costly than the erection of modern hospitals after insanity is established. The time is ripe and the physicians should enlighten the people concerning the genesis of mental disease, its causes and prevention. There is no tangible way of disputing this great law of physical inheritance. The visiting of the iniquities of the fathers upon the children unto the third and fourth generations is the unchallengeable word of the law of God. The law of heredity was intended as a good law and makes evil only when perverted. It was the wisdom of the Infinite Creator that arranged the great plan of propagating the race by the parents imparting their physical equipment to their offspring. Any sacred regard for the parenthood at all would teach the parents to avoid giving their children a diseased and degenerate equipment for life. It is a sad picture to see so many feeble-minded children in our hospitals. They are object lessons of violated law. The Commission of California says: "The ideally normally constructed man is not likely to become insane. Experience teaches that this is strictly true; it also teaches that the child of the weakling will necessarily be a weakling." It is the price men pay in order to satisfy their appetites.

Man in his indulgence of appetite spares not the innocent children who God intended should have a clear heritage. Man should not bring upon those who in coming years are to bear his name everlasting dishonor. Give the youth of the land sound bodies, correct habits and cultured minds. This is the whole duty of man and the conclusion of the whole matter.

"Forty-two and three-tenths per cent of all our insane men and 18 per cent of our insane women had excess in alcohol assigned as the cause of their insanity." This is from Dr. Clouston's report of the Morning Side Asylum in 1903.

Every child has a right to be born healthy and not marked by disease before birth through the sins of its parents. The law should make marriage more difficult and should prohibit lunatics, criminals and habitual drunkards from marriage. If the above classes desire to marry they should be sterilized under the direction of a suitable commission of physicians. If we check the increase of insanity in Texas, we must adopt methods of prevention. With the insane, confirmed criminals and habitual drunkards vasectomy should be compulsory. Sterilization of the above classes has been adopted by some States in the Union—would you think it inhuman for Texas to fall in line?

When the physicians of Texas educate the people along these lines, when they are satisfied that it is not cruel to make defectives unfruitful, which can only be done by physicians, then we will begin to make headway against the encroachment of insanity. We should demand the enactment of such wholesome laws. Humanity demands that we should exercise the same degree of prudence and care in safeguarding the human race that stock breeders exercise in breeding animals.

Dr. Forbes Winslow, of London, says: "Insanity, crime and degeneracy increase in Great Britain in proportion to the amount of alcohol consumed."

